# Sexual dysfunction and quality of life among Tunisian patients with schizophrenia

**DOI:** 10.1192/j.eurpsy.2022.2081

**Published:** 2022-09-01

**Authors:** A. Guermazi, N. Smaoui, D. Jardak, R. Feki, I. Gassara, S. Omri, M. Maalej, L. Zouari, J. Ben Thabet, N. Charfi, M. Maalej

**Affiliations:** Hedi Chaker University Hospital, Sfax, Tunisia, Department Of Psychiatry C, Sfax, Tunisia

**Keywords:** Tunisian patients, sexual dysfunction, Quality of Life, schizophrénia

## Abstract

**Introduction:**

Sexual dysfunction (SD) is prevalent among psychiatric patients than general population.

**Objectives:**

To assess the SD and quality of life (QOL) of patients with schizophrenia, and to identify the factors associated with it.

**Methods:**

This was a cross-sectional, descriptive and analytical study, which began in December 2019, conducted with 60 subjects followed for SCZ or SAD, at the psychiatry outpatient unit of the Hédi Chaker University Hospital in Sfax (Tunisia). General, clinical and therapeutic data were collected using a pre-established questionnaire. The Arizona Sexual Experiences Scale (ASEX) and the 36 item Short-Form Health Survey (SF-36) were used to evaluate subjective sexual dysfunction and QOL respectively.

**Results:**

Patients enrolled had SCZ in 78.2% and SAD in 21.8% of cases. The mean age was 47.2 years. Psychiatric family history, the presence of personal somatic illnesses and tobacco use were found in 43.6%, 61.8% and 67.3% of cases, respectively. The average score of ASEX was 18.21. QOL was altered in 73.3% of participants with an SGM of 53.29. The psychic component was more altered than the physical one with average scores estimated respectively at 48 and 58.44. Participants with SD were more likely to have tobacco consumption (p= 0.025), history of suicide attempt (p=0.023) and they are treated by a combination of several treatments (p=0.025). Impaired QOL was not statistically correlated with SD (p=0.5)

**Conclusions:**

The physicians should pay attention to SD during the assessment and treatment of patients with schizophrenia.

**Disclosure:**

No significant relationships.

